# Identifying factors associated with opioid cessation in a biracial sample using machine learning

**DOI:** 10.37349/emed.2020.00003

**Published:** 2020-02-29

**Authors:** Jiayi W. Cox, Richard M. Sherva, Kathryn L. Lunetta, Richard Saitz, Mark Kon, Henry R. Kranzler, Joel Gelernter, Lindsay A. Farrer

**Affiliations:** 1 Department of Medicine (Biomedical Genetics), Boston University School of Medicine, Boston, MA 02118, USA; 2 Department of Biostatistics, Boston University School of Public Health, Boston, MA 02118, USA; 3 Department of Community Health Sciences, Boston University School of Public Health, Boston, MA 02118, USA; 4 Department of Mathematics and Statistics, Boston University College of Arts & Sciences, Boston, MA 02215, USA; 5 Department of Psychiatry, Perelman School of Medicine, University of Pennsylvania and VISN 4 MIRECC, Crescenz VAMC, Philadelphia, PA 19104, USA; 6 Departments of Psychiatry, Genetics and Neuroscience, Yale School of Medicine, New Haven, CT 06511, USA; 7 Department of Psychiatry, VA CT Healthcare Center, West Haven, CT 06516, USA; 8 Departments of Neurology, Ophthalmology and Epidemiology, Boston University Schools of Medicine and Public Health, Boston, MA 02118, USA

**Keywords:** Opioid use disorder, opioid cessation, machine learning, feature selection, outcome prediction

## Abstract

**Aim:**

Racial disparities in opioid use disorder (OUD) management exist, however, and there is limited research on factors that influence opioid cessation in different population groups.

**Methods:**

We employed multiple machine learning prediction algorithms least absolute shrinkage and selection operator, random forest, deep neural network, and support vector machine to assess factors associated with ceasing opioid use in a sample of 1,192 African Americans (AAs) and 2,557 individuals of European ancestry (EAs) who met Diagnostic and Statistical Manual of Mental Disorders, 5th Edition criteria for OUD. Values for nearly 4,000 variables reflecting demographics, alcohol and other drug use, general health, non-drug use behaviors, and diagnoses for other psychiatric disorders, were obtained for each participant from the Semi-Structured Assessment for Drug Dependence and Alcoholism, a detailed semi-structured interview.

**Results:**

Support vector machine models performed marginally better on average than other machine learning methods with maximum prediction accuracies of 75.4% in AAs and 79.4% in EAs. Subsequent stepwise regression considered the 83 most highly ranked variables across all methods and models and identified less recent cocaine use (AAs: odds ratio (OR) = 1.82, *P* = 9.19 × 10^−5^; EAs: OR = 1.91, *P* = 3.30 × 10^−15^), shorter duration of opioid use (AAs: OR = 0.55, *P* = 5.78 × 10^−6^; EAs: OR = 0.69, *P* = 3.01 × 10^−7^), and older age (AAs: OR = 2.44, *P* = 1.41 × 10^−12^; EAs: OR = 2.00, *P* = 5.74 × 10^−9^) as the strongest independent predictors of opioid cessation in both AAs and EAs. Attending self-help groups for OUD was also an independent predictor (*P* < 0.05) in both population groups, while less gambling severity (OR = 0.80, *P* = 3.32 × 10^−2^) was specific to AAs and post-traumatic stress disorder recovery (OR = 1.93, *P* = 7.88 × 10^−5^), recent antisocial behaviors (OR = 0.64, *P* = 2.69 × 10^−3^), and atheism (OR = 1.45, *P* = 1.34 × 10^−2^) were specific to EAs. Factors related to drug use comprised about half of the significant independent predictors in both AAs and EAs, with other predictors related to non-drug use behaviors, psychiatric disorders, overall health, and demographics.

**Conclusions:**

These proof-of-concept findings provide avenues for hypothesis-driven analysis, and will lead to further research on strategies to improve OUD management in EAs and AAs.

## Introduction

Misuse of illicit and prescription opioids is a significant global problem that affects the health and economic welfare of individuals, families, and society. The U.S. opioid overdose rate has quadrupled since 1991 [1]. In 2017, more than 47,000 Americans died of an opioid overdose, and 36% of these deaths involved prescription opioids [2]. A major goal in treating opioid use disorder (OUD) is abstinence, or complete cessation, of opioid use, other than the use of prescribed opioid replacement therapy. There is not a single, clinically accepted definition of cessation that specifies the length of abstinence required before an individual is no longer considered to have OUD [3, 4]. Diagnostic and Statistical Manual of Mental Disorders, 5th Edition (DSM-5) considers sustained remission from OUD as a one-year period during which no criteria for the disorder (other than craving) are met [5].

Population differences affect multiple aspects of the current epidemic. Although opioid use nationally is higher among individuals of European ancestry (EAs) than African-Americans (AAs), the opioid death rate has increased more sharply among AAs than EAs [6]. AAs have less access to treatment for OUD [7], are less likely to obtain opioid prescriptions for pain management [8], and are incarcerated at a higher rate for illicit opioid use [9] than EAs. Previous research on OUD-related outcomes has been conducted primarily in combined ethnic groups or in EAs only [10], limiting the identification of key population differences in opioid use and treatment outcomes.

Although moderately correlated with opioid cessation, factors contributing to opioid treatment completion such as age, employment status, and age at first drug use have been identified from a mixed ethnicity sample [3]. Other factors are likely to influence cessation, such as pain experiences, general health, and the use of antidepressants [11–13]. Delineation of these factors could inform OUD treatment strategies that may differ across population groups; or could be useful for individuals with OUD who aim to reduce or stop their opioid use. However, studies thus far have tended to focus on a small number of clinically relevant factors such as the dosage, duration, and formulation of medication-assisted treatment of substance use disorders [14–16]. Large epidemiological studies of OUD [17–19] comprised of thousands of variables would allow a systemic, hypothesis-free query to identify factors predicting opioid cessation.

Statistical methods are generally limited in their ability to sort through large numbers of predictors [20]. Data mining using machine learning, which is particularly well suited for identifying predictive factors among thousands of variables [21, 22], has successfully identified predictor variables for a diverse set of outcomes [23–27]. Here, we applied multiple machine learning techniques to evaluate a large set of clinical, demographic, general health, and behavioral variables in a large, racially mixed cohort of individuals who were ascertained for cross-sectional genetic studies of substance use disorders, but not necessarily treated for OUD, to identify factors that are associated with opioid cessation (defined as self-reported last illicit opioid use and/or prescription opioids misuse > 1 year before the interview date). Our study identified additional factors associated with cessation, including several that are population-specific. These findings support an individualized approach to improve the outcome of cessation attempts.

## Materials and methods

### Participants and assessments

Participants for this study were selected from a cohort of 6,188 AAs and 6,835 EAs who were recruited for genetic studies of opioid, cocaine, or alcohol dependence between 2000 and 2017 through advertisements and treatment clinics at Yale University School of Medicine, the University of Connecticut Health Center, the University of Pennsylvania, the Medical University of South Carolina, and McLean Hospital [28, 29]. This cohort included affected sibling pairs and additional family members, as well as unrelated cases and controls. Probands with schizophrenia or bipolar affective disorder were excluded [28, 29]. Information about the use of various substances, demographics, general health, behavior, and other psychiatric illnesses was obtained by interview using the Semi-Structured Assessment for Drug Dependence and Alcoholism (SSADDA) [17, 30]. Substance use disorder (SUD) and psychiatric disorder diagnoses were established according to DSM-IV criteria. Institutional review boards from each recruitment site and Boston University (protocol #H-26819) approved this study, and written informed consent was obtained from all participants.

### Opioid cessation definition

Participants who were eligible for this analysis met at least two DSM-5 criteria for OUD, corresponding to a lifetime diagnosis of OUD. Current opioid cessation was determined by the response to the question, “When was the last time you used an opioid drug (including illicit methadone).” This question was asked as part of a series of items asked about illicit or non-prescribed use of opioids. Individuals who last used an opioid > 1 year before the date of interview were considered to have achieved cessation and those whose last use of an opioid was < 6 months before the interview date were classified as non-cessation. Persons who used opioids between 6 months and 1 year before the interview date were excluded from further analysis. Filtering steps yielding a sample of 1,192 AAs and 2,557 EAs for analysis are shown in Figure S1.

### Phenotype data processing

Preprocessing of 3,956 SSADDA variables was performed prior to machine learning analyses. Variables with narrative or invariable responses, containing redundant information (e.g., specific date of different episodes, drug names), and with a response rate < 90% were removed. Missing values for binary and categorical variables were recoded as indicator variables to accommodate missing responses. Missing values for continuous variables were imputed to the population group mean value. Missing values for ordinal variables related to time since last drug use were assigned the highest level indicating less recent use. Z-score normalization (mean of 0 and variance of 1) was applied to continuous variables within each population to minimize scaling issues. The number of variables remaining after these steps was 3,315 in AAs and 3,738 in EAs.

### Machine learning analyses

AAs and EAs were analyzed separately based on population differences in the epidemiology of opioid use and OUD. Variables were grouped into three nested sets defining three analytical models to explore the prediction accuracy blind to the individual’s opioid or other drug use activities. This approach was adopted to enhance identification of non-drug use variables whose effects may be masked or confounded by variables related to drug use and are highly correlated with the cessation outcome. Model 1 contained all variables except those related to time since last opioid use that are strongly correlated with cessation (variable n = 3,093 in AAs and n = 3,503 in EAs). Model 2 further excluded all opioid-related variables (n = 2,863 in AAs and n = 3,252 in EAs). Model 3 further excluded all drug use variables, leaving only demographic, non-SUD diagnoses and behaviors, and other health-related variables (n = 1,656 in AAs and n = 1,907 in EAs). Models were evaluated using four machine learning methods described in Supplemental Materials to identify variables that are associated with opioid cessation. We modeled different types of inter-variable relationships between potential statistical predictors and the outcome using linear [least absolute shrinkage and selection operator, (LASSO) [31] and linear support vector machine (SVM) with recursive feature elimination (SVM) [32]] and non-linear [random forest (RF) with recursive feature elimination (RF) [33] and deep neural network (DNN) with feature selection (DNN) [34]] techniques. These four methods were applied to capture associated variables under different model assumptions and allow for different outcome-predictor relationships. Variables from each model that were associated with the highest accuracy reflected by either F1 score or area under the curve (AUC) and generated by each machine learning method were retained. The F1 score is a harmonic measure of precision [true positive / (true positive + false negative)] and recall [true positive / (true positive + true negative)], defined by 2 × (precision × recall) / (precision + recall) at a given case/control split, and AUC is an overall evaluation of model performance that accounts for the true positive and false positive rates for all possible diagnostic splits [35, 36]. Both measurements were considered because of their popularity in clinical settings [37]. The F1 score was used to assess accuracy due to limitations of the AUC, which includes bias when performed on imbalanced datasets as well as impractical and uninterpretable split points for evaluation [35, 38].

### Statistical methods for testing the association of opioid cessation with phenotypic variables

To determine which variables selected by the machine learning methods are independently associated with cessation, we applied different cutoffs for the importance measurement of each machine learning method: namely the odds ratio (OR) for LASSO, coefficient [39] denoted by weight for SVM, feature importance [39] for RF, and activation potential [34] for DNN. For LASSO, we chose variables that yielded ORs > 1.05 or < 0.95. We applied the following criteria for selecting variables from SVM and RF analyses depending on the number of variables (*n*) selected for each model: (1) if *n* > 200, the top 30% of variables measured by absolute weight in SVM or importance in RF were designated as high impact, (2) if 100 < *n* < 200, the top 50% were selected, and (3) if *n* < 100, all variables were designated as high impact. For DNN, all selected variables were designated “high impact”. Joint association tests were performed using bi-directional stepwise logistic regression that included 83 “high-impact” variables culled from three models across four machine learning methods in the AA and EA datasets. Variables that yielded the highest Akaike information criterion (AIC) with *P* < 0.05 from bi-directional stepwise logistic regression were grouped into “drug related”, “behavioral”, “other health”, and “demographic” categories.

## Results

Characteristics of the study samples are shown in Table 1. The sample included 1,069 unrelated AAs and 2,252 unrelated EAs, and 123 AA and 305 EA participants who were members of families containing a pair of siblings concordant for opioid or cocaine dependence. There is a higher proportion of females among individuals who ceased opioid use in both AAs (OR = 1.35, *P* = 6.7 × 10^−3^) and EAs (OR = 1.31, *P* = 1.1 × 10^−3^) compared to those who did not cease. Participants who ceased opioid use were also older by an average of 3.18 years in the AA group (*P* = 1.0 × 10^−10^) and 6.1 years in the EA group (*P* = 2.2 × 10^−16^) than those who did not cease use. The mean number of lifetime DSM-5 OUD criteria did not significantly differentiate individuals who ceased opioid use from those who did not.

### Feature selection

The F1 score was generally higher across models in both AAs and EAs using SVM than the other machine learning algorithms (Figure S2), although the differences in F1 score across methods were generally small, especially for models 1 and 2. A detailed discussion of the performance of each method for the three models is provided in Supplemental Materials and Figure S2.

**Figure 1.**
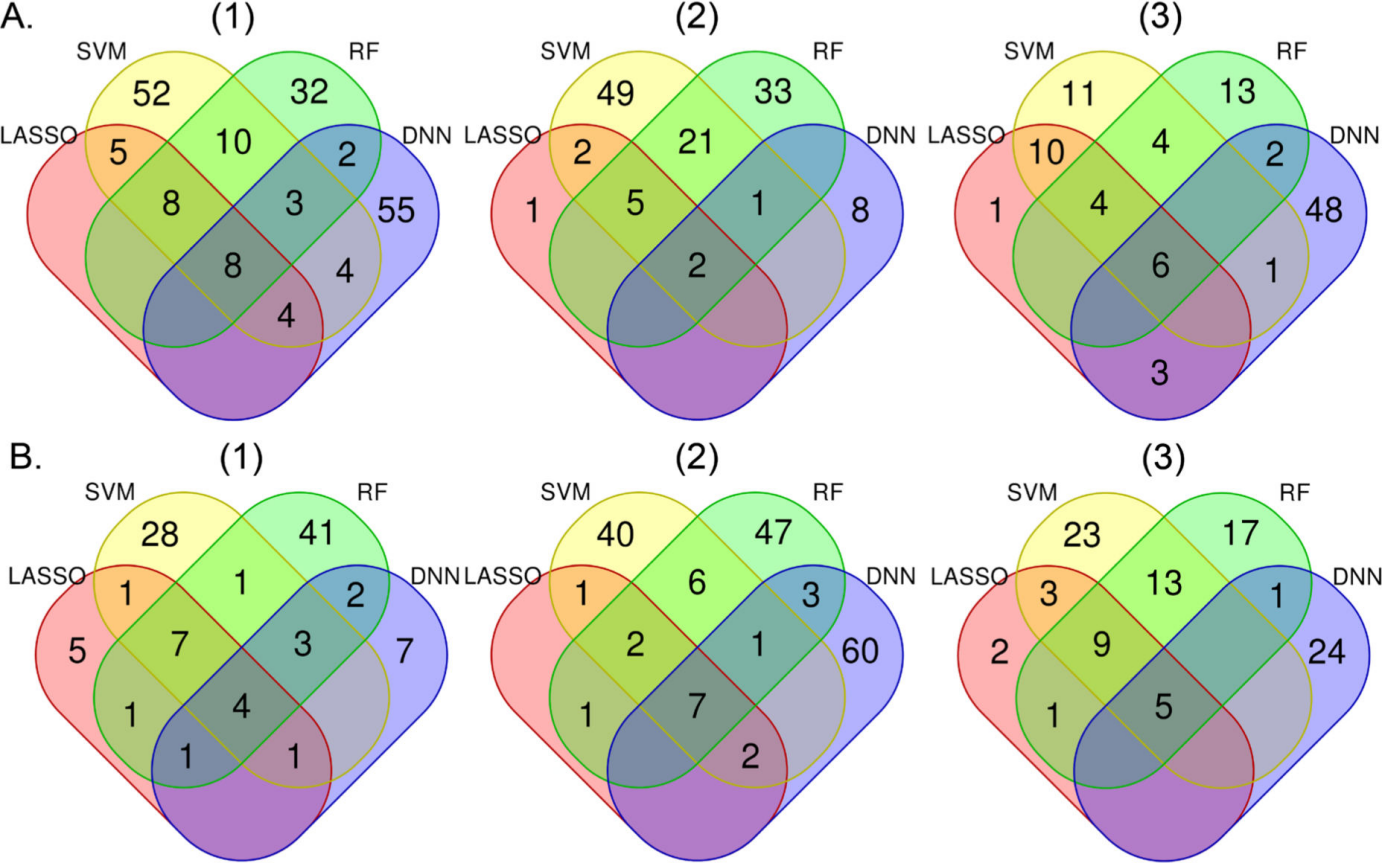
Number of overlapping “high impact” variables selected by each machine learning method based on the importance measurement in African Americans (A) and European Americans (B) for models (1), (2), and (3). Model 1 includes all variables except for those that are confounded with opioid cessation. Model 2 includes all variables in Model 1 except opioid-related variables. Model 3 includes all variables in Model 2 except drug use-related variables. Colors represent particular machine learning methods: pink = LASSO, light yellow = SVM, light green = RF, light blue = DNN

Figure 1 shows the overlap of high impact variables chosen by the four machine learning methods. LASSO “high impact” variables almost entirely overlap with those from the other methods, while DNN-selected variables overlap the least with other method-selected variables. The majority of variables selected by nonLASSO methods are unique to those methods, however, there was high overlap in “high impact” variables selected by SVM and RF. Age was among the five top-ranked variables consistently identified by each method for each model in both AAs and EAs (Table S1). Time since last cocaine use (injection) and recent cocaine use symptoms were selected by all machine learning methods for models 1 and 2 for both AAs and EAs. Also in model 1 analysis, age at first heavy opioid use and years of heroin use were identified in AAs by all machine learning methods. Several variables were selected by all methods in model 2 analyses including time since last cocaine use (injection) in both populations, time since first tobacco use in AAs, and body mass index, age at heaviest weight, and age started heavy cocaine use in EAs. Specific to model 3, positive HIV status, number of children, number of months employed in the last year, and jobless while having drinking and drug problems were selected by all methods in AAs. Under the same model, age at the heaviest weight, co-morbid illnesses, and time since exhibiting last antisocial behavior were selected by all methods in EAs.

### Factors associated with opioid cessation

Stepwise regression analysis that considered 83 “high impact” variables culled from all models and machine learning methods (Table 2, Table S2) identified both population specific factors and non-population specific factors associated with opioid cessation. Variables related to drug use comprised over 50% of the nominally significant predictors of opioid cessation in AAs (29 of 41) and EAs (27 of 50).

Drug related variables were among the most significant positively associated predictors of opioid cessation in AAs and EAs including time since last cocaine injection (OR_AAs_ = 2.30 per level change, *P*_AAs_ = 9.11 × 10^−6^) or use (OR_EAs_ = 1.91 per level change, *P*_EAs_ = 3.30 × 10^−15^), while more years using heroin (OR_AAs_ = 0.55 per standard deviation (SD) change, *P*_AAs_ = 5.78 × 10^−6^) or being older at first heavy opioid use (OR_EAs_ = 0.56 per SD change, *P*_EAs_ = 2.67 × 10^−12^) decreased the odds of the outcome. Several other drug use variables were associated with greater odds of cessation including “more time since last had alcohol symptoms lasting > 1 month” (OR_AAs_ = 1.45 per level change, *P*_AAs_ = 2.84 × 10^−3^) (OR_EAs_ = 1.34 per level change, *P*_EAs_ = 4.13 × 10^−5^), “had 2 marijuana symptoms lasting > 1 month” (OR_AAs_ = 2.13, *P*_AAs_ = 4.83 × 10^−3^) or “marijuana interfered with work or home activities” (OR_EA_ = 1.67, *P*_EA_ = 1.61 × 10^−3^), “smoked less frequently after waking up” (OR_AAs_ = 1.75, *P*_AAs_ = 7.76 × 10^−3^) or the Fagerstrom Test for Nicotine Dependence (FTND) item “able to cut down smoking” (OR_EAs_ = 1.28, *P*_EAs_ = 3.69 × 10^−2^). Having attended a self-help group for OUD (OR_AAs_ = 1.72, *P*_AAs_ = 1.41 × 10^−2^) or started attendance at an OUD self-help group sooner (OR_EAs_ = 1.28 per level change, *P*_EAs_ = 2.4 × 10^−3^) also increased the odds of cessation.

Several variables related to other mental health issues were also associated with opioid cessation. Self-harm (OR_AAs_ = 1.39, *P*_AAs_ = 1.96 × 10^−2^) or suicidal ideation (OR_EAs_ = 1.2, *P*_EAs_ = 8.08 × 10^−3^) were associated with significantly higher odds of cessation. Prior history of a depressive episode lasting > 1 week (OR_AAs_ = 1.31, *P*_AAs_ = 1.66 × 10^−2^) or having drug-use associated depression (OR_EAs_ = 1.64, *P*_EAs_ = 2.55 × 10^−3^) predicted higher odds of cessation. Pathological gambling severity (OR_AAs_ = 0.8, *P*_AAs_ = 3.32 × 10^−2^) and no anxiety for longer than six months (OR_AAs_ = 1.72, *P*_AAs_ = 2.06 × 10^−3^) were significantly associated with cessation in AAs. In EAs, recovering from an event causing post-traumatic stress disorder (PTSD) assessed by the question “no fear in most disturbing/traumatizing event” (OR_EAs_ = 1.93, *P*_EAs_ = 1.66 × 10^−6^), less recent antisocial behavior episodes (OREAs = 1.35 per SD change in age, *P*EAs = 1.03 × 10–4), and unsafely raced cars (OREAs = 1.78, *P*EAs = 3.79 × 10–3) were associated with increased odds of cessation.

Older age was one of the most significantly associated variables with opioid cessation in both population groups (OR_AAs_ = 2.44 per SD change, *P*_AAs_ = 1.41 × 10^−12,^ OR_EAs_ = 2.00 per SD change, *P*_EAs_ = 5.74 × 10^−9^). In AAs, female sex (OR_AAs_ = 1.91, *P*_AAs_ = 1.83 × 10^−3^) and fulltime employment (OR_AAs_ = 1.84, *P*_AAs_ = 1.82 × 10^−2^) were associated with a greater likelihood of opioid cessation, while having been raised primarily by a single parent (OR_AAs_ = 0.63, *P*_AAs_ = 1.3 × 10^−2^) was associated with not achieving cessation. Other variables that were significantly associated with opioid cessation in AAs included HIV positive status (OR = 2.47, *P* = 1.39 × 10^−3^), whereas in EAs higher body mass index (OR = 1.32 per SD change, *P* = 2.58 × 10^−6^), have asthma (OR = 0.68, *P* = 1.22 × 10^−2^), higher household income (OR = 1.15, *P* = 1.3 × 10^−3^), and being an atheist (OR = 1.45, *P* = 1.34 × 10^−2^) were significantly associated with opioid cessation.

## Discussion

We employed both regression and non-regression-based machine learning approaches to evaluate the association of more than 3,000 variables related to SUDs and other psychiatric disorders, other health-related behaviors, and demographic variables with opioid cessation among EAs and AAs assessed in a cross-sectional study of opioid, cocaine, and/or alcohol dependence. We observed moderate-to-high predictive accuracy across all methods; SVM, on average, marginally outperformed the other methods. Although the specific set of associated variables differed in EAs and AAs, a common profile emerged. People who ceased opioid use tended to be older, initiated drug use later in life, had used opioids for a shorter period, experienced fewer problems related to cocaine or alcohol use, were currently employed, and had recovered from other psychiatric disorders including depression and PTSD compared to those whose opioid use persisted.

Previous research using machine learning for addiction outcomes focused mainly on predictive accuracy, although a few studies attempted to identify and interpret specific variables that were associated with the outcomes [3, 4, 40, 41]. Acion et al.[3] reported that ensemble super learning was superior to other machine learning methods, and used penalized regression, SVM, and neural networks for predicting SUD treatment success indicated by treatment discharge status in a Hispanic cohort. In that study, less than 10% of participants had problems with cocaine or illicit opioids and fewer than 30 variables were assessed. In contrast, we evaluated several thousand variables, including detailed measures of drug-use activities and psychiatric disorders, and ranked the importance of the top-ranked variables with four distinct machine learning algorithms. Gowin et al.[40] identified regional brain activity changes predicting relapse from imaging data on fewer than 70 methamphetamine-dependent patients without including any lifestyle factors. Che et al.[4] applied deep learning to electronic health record data to identify people with short-term or long-term opioid use or dependence. Similar to our study, they identified associations with comorbid substance use and anxiety disorders [4]. Several other studies used only regression-based methods to identify variables associated with opioid and stimulant dependence [42], cocaine dependence [43] and alcohol dependence [44], which might not capture other relationships among variables. Several of the non-regression-based methods we employed have also been applied in other studies, which focused mainly on brain magnetic resonance imaging (MRI) traits as predictors of substance use disorder diagnoses [24, 25, 27, 40].

We identified association of opioid cessation with several variables that were previously associated with OUD or OUD-related conditions including co-morbid drug use, antisocial behavior, suicidal thoughts, HIV infection, and asthma [45–50]. Our finding that the majority of people who ceased opioids (60% in AAs and 66% in EAs) also ceased cocaine use is consistent with evidence of high rates of co-occurring OUD and cocaine use disorder (CUD) [51, 52]. This finding also supports the use of treatment strategies that target both disorders [51, 53] and suggests that ceasing use of one substance might influence the ability to cease use of the other. Alternatively, ceasing both opioid and cocaine use may reflect self-selection for inclusion in our genetic studies in which 43% of AA and 32% of EA participants were ascertained for CUD. Our findings are also consistent with observations that a failure to address tobacco use lowers the efficacy of opioid cessation treatment [54] and that a behavioral intervention in patients with antisocial personality disorder reduces substance use [55]. Unlike problems that are associated with other drug use and lower odds of opioid cessation, we found that cannabis use-related problems (e.g., two marijuana symptoms lasting a month, marijuana interfering with work) are associated with higher odds of opioid cessation. This finding is puzzling and not immediately explainable. Although our observation that cannabis users had better success quitting opioids is consistent with prior reports of association of cannabis use and reduced opioid withdrawal symptoms and pain [56, 57], recently we showed that cannabis as a replacement for opioids as treatment strategy for OUD could be harmful [58]. Previous findings of the co-occurrence of drug addiction, suicide attempts, depression, family conflicts, and PTSD, which may suggest bi-directional casual relationships [45–50], are consistent with our observation that better management of comorbid psychiatric problems (fewer recent suicide attempts and psychiatric symptoms) increases the likelihood of opioid cessation or vice versa.

Acion et al.[3] and we identified age, employment status, and age at first drug use as factors for treatment success. The protective effect of older age may be due to ascertainment bias because persons who survived severe dependence are more likely to have stopped using opioids. Full-time employment likely reduces the time or urge for persons dependent on opioids to seek and use the drug. In addition, drug screening associated with some jobs may reduce the likelihood of current opioid use [59]. Quitting opioids also make it easier to find/maintain a job. The inverse correlation of age at first drug use and opioid cessation may reflect the increased difficulty of reversing the effect of long-term opioid exposure on the brain reward system [60] or increased severity associated with earlier onset.

Several variables that were significantly associated with opioid cessation related to non-substance-related behavior were population specific. Although these findings may be due in part to differences between AAs and EAs in willingness to endorse these behaviors, previous studies showed that AAs were more likely than EAs to report prolonged gambling and problems associated with gambling [61, 62]. One explanation for our findings of significant associations of ceasing opioids with a self-reported HIV diagnosis in AAs is that OUD patients with severe or life-threatening illnesses are more likely to seek or adhere to treatment [63], an idea supported by evidence that HIV-infected patients have better treatment outcomes for OUD [64–66]. Alternatively, poorer general health may lead to reduced drug use [67] (the so-called “sick quitter”). In contrast, antisocial behavior, recovery from PTSD, and being an atheist were associated with opioid cessation in EAs only. Prior research may provide insight into these EA-specific patterns. One study reported antisocial behaviors in EA children were significantly associated with substance initiation while the association was less strong in AA children [68], although the impact on opioid use was not assessed. PTSD and being an atheist identified in EAs might be due to the racial difference in exposure to traumatic events and belief diversities [69, 70]. Previous evidence about the effect of religion on SUDs is contradictory. One study showed that loss of religiosity between childhood and adulthood was associated with increased substance use while recent religiosity increased the odds of illicit drug use in the past year [71]. Alternatively, the smaller sample of AAs might have limited our ability to detect these associations in that group.

The current study has several strengths. First, because the input dataset contains thousands of variables related to drug use activities, psychiatric disorders, medical history, and demographics obtained from several thousand individuals meeting DSM-5 criteria for OUD, we were able to explore many factors in addition to those included in other studies. Second, both linear and non-linear machine learning methods were employed to model the true underlying relationship between the variables and outcome, which increased the number of factors we identified. Third, we evaluated three models for each machine learning method in order to better understand the contribution of opioid and other drug use information. Fourth, we considered only independent variables in the association analyses to prevent over-representation of correlated factors. Finally, although there is no published “gold standard” predictive model against which to compare our results, the 80% predictive accuracy we achieved is similar to that seen in other machine learning studies [3, 4, 40, 41].

Limitations of this work should be noted. First, given the cross-sectional nature of our data and the over 90% relapse rate for OUD [72], many individuals classified as not using opioids may have subsequently relapsed to opioid use. However, it has been shown that prior abstinence is predictive of future abstinence, therefore people who ceased opioids are more likely to cease again even when relapse occurs [73]. Second, the machine learning analyses were based on samples that may have been underpowered to detect associations with some variables compared to other studies that included tens of thousands of individuals [74]. Third, most persons in our cohort were evaluated prior to the current opioid epidemic and may not reflect recent secular trends in the prevalence and associated features of OUD. Fourth, associations of cessation with some variables and overall prediction accuracy may have been inflated because our analysis did not fully account for familial correlations. Fifth, in spite of the large number of variables that were included in the machine learning analyses, potentially important variables such as the reasons for first use and details of treatment and support programs were unavailable. However, we identified attending an opioid addiction self-help group as associated with successful cessation, which is consistent with the reported benefit of self-help groups [75]. Sixth, the rate of response to many interview questions was substantially higher in EAs, while the sample size was twice that of AAs, which could account for some of the observed racial differences in predictive models. Related to this concern, our use of mean imputation for missing data may have been overly conservative. Other methods, including cold/hot deck (such as K nearest neighbors) and multiple imputation [76], may have provided additional information but were not appropriate for this dataset given its size, use of continuous, ordinal, and binary variable coding, non-linear relationships among variables, and lack of an appropriate external reference. Seventh, given the limited amount of temporal information with respect to many of the potential risk factors for opioid cessation in this cross-sectional sample, it is difficult to determine the effect direction for many of the observed associations. Because of these limitations, our findings require external validation in larger samples before they can be incorporated in prediction models for clinical purposes. Finally, while some of the factors we identified are plausible and consistent with prior studies, other factors such as atheism are not immediately interpretable. Thus, because our research is atheoretical, results should be interpreted with caution and be validated before implemented in clinical practice.

In conclusion, we analyzed a large number of variables including demographic, behavioral, health and drug use activities using machine learning techniques with feature selection and found variables in a wide range of domains that were associated with cessation. These included some that are consistent with prior literature, plausible but have not been well studied, and do not have readily apparent explanations for their associations. Our findings suggest hypotheses for future studies and could inform how one might increase the likelihood of cessation with and without treatment. These results also support several widely known treatment strategies for OUD, such as treating psychiatric comorbidity, adding wraparound services such as employment counseling, and simultaneously addressing polydrug use problems. Finally, in an era of increasing availability of digitized health-related records, our study provides a framework for disease outcome prediction using high dimensional phenotypic data collected via a research instrument.

## Supplementary Material

Supplemental Methods and Table

## Figures and Tables

**Table 1.** Participant characteristics

		Time since last use	
		≤ 6 month (not cease)	> 1 year (ceased)

AAs (*N* = 1192)	Number (% female)	701 (33.5%)	491 (40.5%)
	Age (Mean ± SD)	42.6 ± 8.5	45.6 ± 8.3
	OUD Symptom Counts (Mean ± SD)	7.8 ± 2.4	7.6 ± 2.5
	Number of families (number in families)	35 (76)	23 (47)
EAs (*N* = 2557)	Number (% female)	1714 (34.4%)	843 (40.6%)
	Age (Mean ± SD)	34.4 ± 10	40.5 ± 10.3
	OUD symptom Counts (Mean ± SD)	8.8 ± 1.9	8.4 ± 2.3
	Number of families (number in families)	114 (241)	31 (64)

SD: standard deviation

**Table 2.** Variables associated with opioid cessation at *P* < 0.01 in the (A) African American and (B) European ancestry groups

**(A) African American**			
	**Variable**	**OR**	***P*-value**
Drug related	Time since 1st opioid treatment*	1.56	1.90E-04
	Older age at first opioid symptoms^$^	0.46	2.23E-05
	Number of years using heroin^$^	0.55	5.78E-06
	Depressed after reducing cocaine use	0.53	4.05E-03
	Time since last injected cocaine*	2.30	9.11E-06
	Time since last used cocaine*	1.82	9.19E-05
	Time since last stayed high in cocaine*	1.41	2.93E-03
	Used cocaine < 11 times within year of interview	2.67	1.38E-03
	Treated in outpatient program for cocaine use	1.88	4.06E-03
	Time since of first cocaine craving*	0.71	1.59E-03
	Never injected cocaine	2.53	1.75E-03
	Often used marijuana more than intended to	0.40	5.67E-04
	Mixed alcohol and drugs > 3 times in 12 months	0.51	2.08E-03
	Time since last had alcohol symptoms lasting > 1 month*	1.45	2.84E-03
	Smoked less frequently after waking up	1.75	7.76E-03
	Older age at first cigarette^$^	1.31	6.24E-03
	Had 2 marijuana symptoms lasting a month	2.13	4.83E-03
Other Health	HIV positive	2.47	1.39E-03
	Health has always been better than now	0.62	9.64E-03
Demographic	Female sex	1.91	1.83E-03
	Current age	2.44	1.41E-12
**(B) European ancestry**			
	**Variable**	**OR**	***P*-value**
Drug related	Time since last cocaine use*	1.91	3.30E-15
	Older age at first heavy opioid use^$^	0.56	2.67E-12
	Number of years using heroin^$^	0.69	3.01E-07
	Time since last cocaine injection*	1.85	2.38E-06
	Time since last had alcohol symptoms that last > 1 month*	1.34	4.13E-05
	> 20 outpatient visits for drug/psychiatric problems in the last year	1.76	9.56E-06
	Time since opioid treatment initiation*	1.53	2.24E-06
	Used cocaine > 11 times in last year	0.47	1.09E-05
	Time since first used opioid 1/week for > 1 month*	1.41	1.10E-04
	Have injected cocaine	2.01	1.66E-04
	Older age at first heavy cocaine use^$^	1.27	7.40E-04
	Marijuana interfered with work/home	1.67	1.61E-03
	Time since one started opioid self-help group*	1.28	2.40E-03
	Time since last feel high on cocaine for > 1 day*	1.33	1.15E-03
	Time since last attended cocaine self-help group*	0.76	3.27E-04
	Used tobacco but not addicted	0.60	3.72E-03
	Disclosed problems with cocaine usage to professional	1.65	2.45E-03
	Stopped using stimulants for > 3 month	1.99	2.38E-03
	Drinking resulted in objections or problems with family and work	1.48	4.39E-03
Behavioral	< 3 ASP criteria in 12-month period	0.64	2.69E-03
	Often failed to pay debts	0.68	2.17E-03
	Suspended or expelled from school	0.67	2.16E-03
	Time since last had suicidal ideation*	1.20	8.08E-03
	Less recent since last had antisocial behaviors^$^	1.35	1.03E-04
	No fear of most disturbing/traumatizing event	1.93	1.66E-06
	Avoided scenes that reminded of traumatic event	1.88	7.88E-05
	Had OCD behaviors when depressed	0.49	3.23E-04
	Feeling distracted	1.56	8.15E-04
	Unsafely raced cars	0.56	3.79E-03
	Depression always started with drug problems	1.64	2.55E-03
	Number of depression symptoms	1.46	8.99E-03
	Have outstanding emotional problem	1.63	5.55E-03
Other Health	Body mass index	1.32	3.59E-06
Demographics	Household income	1.15	1.30E-03
	Current age	2.00	5.74E-09

OR = odds ratio.

* Categorical variable: 1 = within the last two weeks, 2 = two weeks to less than one month ago, 3 = one month to less than six months ago, 4 = six months to one year ago, 5 = more than a year ago. OR represents the factor increase per level change.

$ Continuous variable: OR represents the factor increase per standard deviation unit

## Abbreviations

AA: African American
AIC: Akaike information criterion
AUC: area under the curve CUD: cocaine use disorder
DNN: deep neural network
DSM-5: Diagnostic and Statistical Manual of Mental Disorders, 5th Edition
EA: European ancestry
FTND: Fagerstrom Test for Nicotine Dependence
LASSO: least absolute shrinkage and selection operator
MRI: magnetic resonance imaging
OR: odds ratio
OCD: obsessive-compulsive disorder
OUD: opioid use disorder
PTSD: post-traumatic stress disorder
RF: random forest
SD: standard deviation
SSADDA: Semi-Structured Assessment for Drug Dependence and Alcoholism
SUD: substance use disorder
SVM: support vector machine
