# Supplementary material for: Identifying factors associated with opioid cessation in a biracial sample using machine learning
Source: Explor Med. Author manuscript; Available in PMC 2021 Feb 4. (PMC7861053; doi:10.37349/emed.2020.00003)
Supplement: Supplemental Methods and Table [file NIHMS1580456-supplement-Supplemental_Methods_and_Table.docx]

**Supplemental Methods**

Least Absolute Shrinkage and Selection Operator (LASSO)

LASSO implemented in the R package ‘glmnet’ (1) was used for both feature selection and prediction. The shrinkage parameter lambda in the penalty term of LASSO regression was obtained using 10-fold cross validation on the training set 10 times. Separate accuracy criteria of either misclassification error or AUC were used to search for the lambda with the best model fit. The “1SE rule (2)” which aims to find the simplest model with comparable accuracy to the best model, was used to identify lambda whose cross validation error was one standard error unit from the lowest cross validation error on the training set. We identified the significant features by fitting lambdas on the training set. The test set accuracy was evaluated by using the class probability prediction of test set as an input to Scikit-learn (3) (roc_auc_score and f1_score) to obtain our final accuracy measures: F1 score and AUC.

Support Vector Machine (SVM) with Recursive Feature Elimination

Linear SVM with recursive feature elimination was implemented using the Scikit-learn python package. A soft margin was included in the model to reduce overfitting. Briefly, we set the penalty parameter C by exhaustively searching the range of values between 2^-20^ and 2^8^ that covers the range recommended by Hsu et.al (4) using 10-fold cross validation with balanced class weights on the training set . We applied the parameter C chosen by either maximizing AUC or F1 score (whichever yielded the highest accuracy) in the training set for recursive feature selection. A feature’s importance, represented by its weight, was used as the input to the recursive feature elimination function, where 10-fold cross validation was used to find the combination of features that maximizes either AUC or F1 score (whichever yielded the highest accuracy). Selected features from each model were applied to the test set to obtain the overall test accuracy.

Random Forest (RF) Recursive Feature Elimination

The RF recursive feature elimination approach was implemented using the Scikit-learn python package. We determined the ideal number of decision trees needed for the RF model by searching the range of 2^7^ to 2^11^ recommended by Oshiro et.al (5) 10-fold cross validation with balanced class weight on the training set. The decision trees that associated with the maximum AUC or maximum F1 score were individually chosen. A feature’s importance, represented by the Gini impurity (6, 7), was used to evaluate the importance of a variable by adding up the weighted impurity decreases for all nodes averaged over all decision trees. A feature’s importance rank was used as the input for recursive feature elimination and 10-fold cross validation was used to find the combination of features that maximized either AUC or F1 score (whichever yielded the highest accuracy). Selected features from each model were included in the test set without any feature selection steps to obtain the test accuracy.

Deep Neural Network (DNN)

The DNN approach was applied using Keras (8) with a tensorflow framework. A 3-layer, fully connected feed forward DNN was constructed with two hidden layers using rectified linear units (Relu) as an activation function and a sigmoid function for the output layer. The training set that was used in the aforementioned methods was further split into a development set and validation set with 9:1 ratio with fixed case control ratios to minimize overfitting or inadequate training. The Adam optimization method was used to find efficiently the parameters associated with the ideal state of the DNN objective function. The Adam optimizer (9) was implemented using the “state of the art” optimization approach (10-12) by incorporating the advantages of two popular optimizations (RMSProp (13) and AdaGrad (14). We followed the suggested hyper-parameter settings (9) and tuned only the learning rate. L2 normalization with various scales was performed on each layer to prevent overfitting. Cross validation of the development set and balanced accuracy were used to reduce bias for the hyper-parameter search. The weighted AUC and weighted F1 score on the validation set were used to measure model performance.

Several methods have been proposed for feature selection using DNN, with the focus on reducing input dimensionality, such as sparse one-to-one, dropout feature ranking, and activation potential based (15-17). We used the activation potential based method because of its proven performance in reducing the number of features, to obviate application of another filtering method coupled with DNN, and its simplicity and intuitiveness for selecting the number of important variables. Feature selection was performed according to the method proposed by Roy et.al (17). Briefly, we computed the activation potential of each input feature connected to each of the hidden nodes in the first layer before applying Relu. Then, the average relative activation potential of each feature in each first hidden layer node was calculated by averaging the number of input features and training samples at each node. Relu activation was applied to the average relative activation potential to obtain the net positive contribution of each input feature. Input features were ranked and plotted against their net positive activation potential contribution. Important features were chosen based on their collective contribution of net positive activation potential. Because of bias associated with AUC in the presence of imbalanced dataset, the feature combination that associated with the highest F1 score in the validation set was used to obtain the accuracy on test set.

Performance of Machine Learning Algorithms

There was a consistent drop in accuracy from model 1 to model 3 across the four machine learning methods, although the difference between models 1 and 2 is smaller than the difference between models 2 and 3 (Figure S2). The loss of accuracy across models was greater in AAs than in EAs. The AUC generally demonstrated higher accuracy than the F1 score. Among the machine learning methods, SVM yielded the highest F1 score more frequently than the other methods across models in both AAs and EAs. Most notably, SVM had the best performance for model 2 in AAs and models 2 and 3 in EAs, although the differences in accuracy between SVM and the other models with high performance were small. The observation that SVM performed only marginally better than LASSO in both AAs and EAs was not surprising because SVM using a linear kernel and LASSO employ a linear model with regularization. Both SVM and LASSO selected uncorrelated features, however SVM also selected correlated features. Both RF and DNN showed some evidence for overfitting (result not shown), although the effect was relatively small that was reflected by an approximately 4% higher accuracy in the cross validation training set than the test set. The RF model may have been over-fitted because the number of individual classifier decision trees was fixed. DNN generally requires a much larger sample size than the one available here, which might have limited its performance.

**Supplemental Table 1**. Five most significant variables ranked by feature importance and their p values from stepwise regression considering all variables for each machine learning method, stratified by model and population.

**African Americans**

|  |  | Variable | Rank | p-value |
| --- | --- | --- | --- | --- |
| Model 1 | LASSO | Time since last cocaine use | 1 | 2.89E-07 |
|  |  | Current age | 2 | 5.39E-10 |
|  |  | Time since 1st opioid treatment | 3 | 4.97E-06 |
|  |  | Cocaine use severity | 4 | 4.69E-03 |
|  |  | Number of years using heroin | 5 | 2.80E-08 |
|  | SVM | Time since last cocaine use | 1 | 3.50E-08 |
|  |  | Time since last cocaine injection | 2 | 1.38E-04 |
|  |  | Current age | 3 | 9.20E-07 |
|  |  | Number of years using heroin | 4 | 7.68E-06 |
|  |  | Time since weekly cocaine use for 1 month | 5 | NS |
|  | RF | Time since last cocaine use | 1 | 4.38E-06 |
|  |  | Time since weekly cocaine use for 1 month | 2 | NS |
|  |  | Used cocaine>11 times in the last year | 3 | NS |
|  |  | Recently had >1 cocaine symptoms | 4 | NS |
|  |  | Recently had >2 cocaine symptoms | 5 | NS |
|  | DNN | Time since last cocaine use | 1 | 1.04E-06 |
|  |  | Time since last cocaine injection | 2 | NS |
|  |  | Recently had >1 cocaine symptoms | 3 | 1.11E-01 |
|  |  | Current age | 4 | 5.50E-08 |
|  |  | Time since weekly cocaine use for 1 month | 5 | NS |
| Model 2 | LASSO | Time since last cocaine use | 1 | 5.32E-15 |
|  |  | Current age | 2 | 3.96E-08 |
|  |  | Cocaine use severity | 3 | 1.24E-07 |
|  |  | Time since last tobacco use | 4 | 1.62E-03 |
|  |  | Recently reported drug problems to professionals | 5 | 9.65E-01 |
|  | SVM | Time since last cocaine use | 1 | 5.44E-07 |
|  |  | Time since weekly cocaine use for 1 month | 2 | NS |
|  |  | Recently had >1 cocaine symptoms | 3 | NS |
|  |  | Used use cocaine>11 times last year | 4 | 2.61E-02 |
|  |  | Current age | 5 | 4.69E-08 |
|  | RF | Time since last cocaine use | 1 | 2.26E-06 |
|  |  | Used cocaine>11 times last year | 2 | 4.38E-02 |
|  |  | Recently had >1 cocaine symptoms | 3 | NS |
|  |  | Time since weekly cocaine use for 1 month | 4 | NS |
|  |  | Recently had >2 cocaine symptoms | 5 | NS |
|  | DNN | Age first used tobacco | 1 | 9.42E-04 |
|  |  | Had romantic relationship for >1 year | 2 | 6.54E-03 |
|  |  | Longest time in days without using tobacco | 3 | NS |
|  |  | Contacted relatives infrequently | 4 | NS |
|  |  | Mixed drugs with alcohol >3 times | 5 | NS |
| Model 3 | LASSO | Current age | 1 | 5.20E-11 |
|  |  | HIV positive | 2 | 8.55E-06 |
|  |  | Blamed others for one's mistake | 3 | 1.76E-03 |
|  |  | Jobless for > 6 months due to drugs/alcohol | 4 | 2.93E-02 |
|  |  | Currently unemployed | 5 | 2.97E-04 |
|  | SVM | Current age | 1 | 2.67E-09 |
|  |  | Number of biological children | 2 | 3.62E-02 |
|  |  | Currently unemployed | 3 | 3.94E-02 |
|  |  | Age at maximum weight | 4 | NS |
|  |  | Number of months employed last year | 5 | NS |
|  | RF | Current age | 1 | 2.49E-09 |
|  |  | Age at maximum weight | 2 | NS |
|  |  | Body mass index | 3 | 5.98E-06 |
|  |  | Number of drug symptoms when depressed | 4 | 1.13E-02 |
|  |  | Current weight | 5 | NS |
|  | DNN | Number of doctor visits for health problems | 1 | NS |
|  |  | Current age | 2 | 5.17E-11 |
|  |  | Jobless for > 6 months due to drugs/alcohol | 3 | 1.49E-01 |
|  |  | Treated for emotional, psychiatric or drug problems | 4 | 3.51E-03 |
|  |  | Visited treatment center once last year | 5 | 1.25E-01 |

**European Americans**

|  |  | Variable | Rank | p-value |
| --- | --- | --- | --- | --- |
| Model 1 | LASSO | Time since last cocaine use | 1 | 8.72E-19 |
|  |  | Current age | 2 | 9.96E-12 |
|  |  | Time since starting opioid treatment | 3 | 9.31E-07 |
|  |  | Time since last cocaine injection | 4 | 1.80E-04 |
|  |  | Age last had antisocial behaviors | 5 | 3.92E-06 |
|  | SVM | Time since last cocaine use | 1 | 1.01E-16 |
|  |  | Time since last cocaine injection | 2 | 1.01E-06 |
|  |  | Used cocaine>11 times in last year | 3 | 3.10E-03 |
|  |  | Age at maximum weight | 4 | 5.46E-02 |
|  |  | Current age | 5 | 9.87E-03 |
|  | RF | Time since last cocaine use | 1 | 4.38E-06 |
|  |  | Recently had >1 cocaine symptoms | 2 | 3.24E-03 |
|  |  | Use cocaine>11 times in last year | 3 | 2.79E-03 |
|  |  | Time since last cocaine injection | 4 | 6.52E-03 |
|  |  | Recently had >2 cocaine symptoms | 5 | NS |
|  | DNN | Time since last cocaine use | 1 | 4.79E-25 |
|  |  | Recently disclosed opioid problems with professionals | 2 | NS |
|  |  | Time since last cocaine injection | 3 | 1.07E-03 |
|  |  | Age last smoked cigarettes | 4 | 6.60E-21 |
|  |  | Drunk driving arrests (y/n) | 5 | NS |
| Model 2 | LASSO | Time since last cocaine use | 1 | 3.06E-18 |
|  |  | Current age | 2 | 1.27E-05 |
|  |  | Time since last cocaine injection | 3 | 1.38E-08 |
|  |  | Age last had antisocial behaviors | 4 | 4.56E-06 |
|  |  | Cocaine use severity | 5 | 1.83E-07 |
|  | SVM | Time since last cocaine use | 1 | 3.14E-17 |
|  |  | Time since last cocaine injection | 2 | 1.74E-06 |
|  |  | Used cocaine>11 times last year | 3 | 8.36E-05 |
|  |  | Age at maximum weight | 4 | 3.82E-02 |
|  |  | Current age | 5 | 1.64E-05 |
|  | RF | Time since last cocaine use | 1 | 3.13E-14 |
|  |  | Time since last cocaine injection | 2 | 2.43E-06 |
|  |  | Used cocaine>11 times last year | 3 | 8.16E-04 |
|  |  | Recently had >1 cocaine symptoms | 4 | NS |
|  |  | Current age | 5 | 5.89E-06 |
|  | DNN | Time since last cocaine use | 1 | 1.42E-17 |
|  |  | Hurt animal on purpose | 2 | NS |
|  |  | Recently last stayed high from cocaine >1 day | 3 | 2.83E-03 |
|  |  | Recently had >2 cocaine symptoms | 4 | NS |
|  |  | Recently had >1 cocaine symptoms | 5 | NS |
| Model 3 | LASSO | Current age | 1 | 2.36E-09 |
|  |  | Current health has always been worse | 2 | 7.62E-07 |
|  |  | Body mass index | 3 | 3.12E-13 |
|  |  | Age last had antisocial behaviors | 4 | NS |
|  |  | Depression started with drug problems | 5 | 3.98E-03 |
|  | SVM | Current age | 1 | 3.10E-08 |
|  |  | Age at maximum weight | 2 | 4.62E-03 |
|  |  | Age last had antisocial behaviors | 3 | 2.74E-07 |
|  |  | Body mass index | 4 | 5.89E-12 |
|  |  | Current weight | 5 | NS |
|  | RF | Current age | 1 | 5.10E-09 |
|  |  | Age at maximum weight | 2 | 2.36E-02 |
|  |  | Body mass index | 3 | 1.15E-04 |
|  |  | Current weight | 4 | 8.08E-02 |
|  |  | Maximum weight | 5 | 4.51E-03 |
|  | DNN | Current age | 1 | 2.13E-09 |
|  |  | Bad mood after ECT or bright light therapy | 2 | NS |
|  |  | Have OCD | 3 | NS |
|  |  | Had gambling withdrawal when cannot gamble | 4 | 9.20E-02 |
|  |  | Had sex with >10 people in a year | 5 | 3.54 E-02 |

Rank = relative importance of a variable measured by the specified method; NS = variable not selected by stepwise regression; LASSO = least absolute shrinkage and selection operator; SVM = support vector machine with recursive feature elimination; RF = random forest with recursive feature elimination; DNN = deep neural network with recursive feature elimination.

**Supplemental Table 2**. Variables associated with opioid cessation at P<0.05 in (A) African Americans and (B) European Americans.

**A. African Americans**

|  | **Variable** | **OR** | **p-value** |
| --- | --- | --- | --- |
| Drug related | Time since 1st opioid treatment^*^ | 1.56 | 1.90E-04 |
|  | Older age at first opioid misuse^$^ | 1.4 | 2.45E-02 |
|  | Older age at first opioid symptoms^$^ | 0.46 | 2.23E-05 |
|  | Diarrhea after stopping opioid use | 1.54 | 3.68E-02 |
|  | Attended opioid self-help group | 1.72 | 1.41E-02 |
|  | Older age at first heavy use of opioids^$^ | 0.73 | 2.70E-02 |
|  | N years using heroin^$^ | 0.55 | 5.78E-06 |
|  | Depressed after reducing cocaine use | 0.53 | 4.05E-03 |
|  | Time since last injected cocaine^*^ | 2.30 | 9.11E-06 |
|  | Time since last used cocaine^*^ | 1.82 | 9.19E-05 |
|  | Time since last stayed high in cocaine^*^ | 1.41 | 2.93E-03 |
|  | Used cocaine <11 times within year of interview | 2.67 | 1.38E-03 |
|  | Treated in outpatient program for cocaine use | 1.88 | 4.06E-03 |
|  | Time since of first cocaine craving^*^ | 0.71 | 1.59E-03 |
|  | Never injected cocaine | 2.53 | 1.75E-03 |
|  | Often used marijuana more than intended to | 0.40 | 5.67E-04 |
|  | Mixed alcohol and drugs >3 times in 12 months | 0.51 | 2.08E-03 |
|  | Time since last had alcohol symptoms lasting >1 month^*^ | 1.45 | 2.84E-03 |
|  | Drinking interfered with work/responsibility | 0.60 | 2.71E-02 |
|  | Time since last attended alcohol self-help group^*^ | 0.79 | 2.65E-02 |
|  | Being alcohol dependent | 1.73 | 2.56E-02 |
|  | Smoked in dangerous situation >3 times | 0.66 | 3.75E-02 |
|  | Smoked less frequently after waking up | 1.75 | 7.76E-03 |
|  | Older age at first cigarette^$^ | 1.31 | 6.24E-03 |
|  | Gave up social activities because of smoking | 1.82 | 1.73E-02 |
|  | Time since first had sedatives^*^ | 1.3 | 1.06E-02 |
|  | Had 2 marijuana symptoms lasting a month | 2.13 | 4.83E-03 |
|  | Disclosed drug problems to professionals | 1.53 | 2.00E-02 |
|  | Number of years using sedatives^$^ | 1.29 | 1.33E-02 |
| Behavioral | Pathological gambling severity | 0.80 | 3.32E-02 |
|  | Time since last hurt oneself on purpose^*^ | 1.39 | 1.96E-02 |
|  | Time since last had depression >1 week^*^ | 1.31 | 1.66E-02 |
|  | Ever treated with medication or ECT for depression | 1.75 | 4.33E-02 |
|  | Heard delusional noises when awake | 1.61 | 3.40E-02 |
|  | No anxiety for >6 months | 1.72 | 2.06E-02 |
| Other Health | HIV positive | 2.47 | 1.39E-03 |
|  | Health has always been better than now | 0.62 | 9.64E-03 |
| Demographic | Female sex | 1.91 | 1.83E-03 |
|  | Raised primarily by single parent | 0.63 | 1.30E-02 |
|  | Current age | 2.44 | 1.41E-12 |
|  | Fulltime employment | 1.84 | 1.82E-02 |

**B. European Americans**

|  | **Variable** | **OR** | **p-value** |
| --- | --- | --- | --- |
| Drug related | Time since last cocaine use^*^ | 1.91 | 3.30E-15 |
|  | Older age at first heavy opioid use^$^ | 0.56 | 2.67E-12 |
|  | Number of years using heroin^$^ | 0.69 | 3.01E-07 |
|  | Time since last cocaine injection^*^ | 1.85 | 2.38E-06 |
|  | Time since last had alcohol symptoms that last >1 month^*^ | 1.34 | 4.13E-05 |
|  | >20 outpatient visits in past year for drug/psychiatric problems | 1.76 | 9.56E-06 |
|  | Time since opioid treatment initiation^*^ | 1.53 | 2.24E-06 |
|  | Used cocaine >11 times in last year | 0.47 | 1.09E-05 |
|  | Time since first used opioid 1/week for >1 month^*^ | 1.41 | 1.10E-04 |
|  | Have injected cocaine | 2.01 | 1.66E-04 |
|  | Older age at first heavy cocaine use^$^ | 1.27 | 7.40E-04 |
|  | Marijuana interfered with work/home | 1.67 | 1.61E-03 |
|  | Time since one started opioid self-help group^*^ | 1.28 | 2.40E-03 |
|  | Time since last feel high on cocaine for >1 day^*^ | 1.33 | 1.15E-03 |
|  | Time since last attended cocaine self-help group^*^ | 0.76 | 3.27E-04 |
|  | Continued using stimulant for its psychological problems | 0.54 | 2.27E-02 |
|  | Heart slowed down when cutting down tobacco use | 0.56 | 1.52E-02 |
|  | Used tobacco but not addicted | 0.60 | 3.72E-03 |
|  | Disclosed problems with cocaine usage to professional | 1.65 | 2.45E-03 |
|  | Always able to cut down smoking | 1.28 | 3.69E-02 |
|  | Treated at outpatient drug program for cocaine | 1.41 | 2.81E-02 |
|  | Used opioid more than intended to | 0.63 | 1.15E-02 |
|  | Craved cocaine when cut down | 1.42 | 1.05E-02 |
|  | Maximum N drinks in 24 hours^$^ | 1.15 | 1.61E-02 |
|  | Ever smoked >1 pack of cigarette daily for >1 month | 1.16 | 2.16E-02 |
|  | Stopped using stimulants for >3 month | 1.99 | 2.38E-03 |
|  | Drinking resulted in problems with family and work | 1.48 | 4.39E-03 |
| Behavioral | Answered questions before they were completed as a child | 0.73 | 2.24E-02 |
|  | <3 ASP criteria in 12 month period | 0.64 | 2.69E-03 |
|  | Often failed to pay debts | 0.68 | 2.17E-03 |
|  | Suspended or expelled from school | 0.67 | 2.16E-03 |
|  | Number times in jail^$^ | 0.85 | 1.05E-02 |
|  | Time since last had suicide idea^*^ | 1.20 | 8.08E-03 |
|  | Less recent since last had antisocial behaviors^$^ | 1.35 | 1.03E-04 |
|  | No fear of most disturbing/traumatizing event | 1.93 | 1.66E-06 |
|  | Avoided scenes that reminded of traumatic event | 1.88 | 7.88E-05 |
|  | Had OCD behaviors when depressed | 0.49 | 3.23E-04 |
|  | Feeling distracted | 1.56 | 8.15E-04 |
|  | Unsafely raced cars | 0.56 | 3.79E-03 |
|  | Found customers for prostitutes | 0.68 | 4.04E-02 |
|  | Depression always started with drug problems | 1.64 | 2.55E-03 |
|  | Number of depression symptoms | 1.46 | 8.99E-03 |
|  | Time since last had depression episode^*^ | 1.20 | 1.65E-02 |
|  | Have outstanding emotional problem | 1.63 | 5.55E-03 |
| Other Health | Have asthma | 0.68 | 1.22E-02 |
|  | Being older at heaviest weight^$^ | 1.21 | 2.68E-02 |
|  | Body mass index | 1.32 | 3.59E-06 |
| Demographic | Being an atheist | 1.45 | 1.34E-02 |
|  | Household income | 1.15 | 1.30E-03 |
|  | Current age | 2.00 | 5.74E-09 |

OR=odds ratio

* Categorical variable: 1 = within the last two weeks, 2 = two weeks to less than one month ago, 3 = one month to less than six months ago, 4 = six months to one year ago, 5 = more than a year ago. OR represents the factor increase per level change.

$ Continuous variable: OR represents the factor increase per standard deviation unit.

**Supplemental Figure 1**. Derivation of African Americans (AAs) and European Americans (EAs) subjects in the Yale-Penn dataset who were ascertained from multiple substance use disorder studies and met criteria for cessation or non-cessation of opioid use.


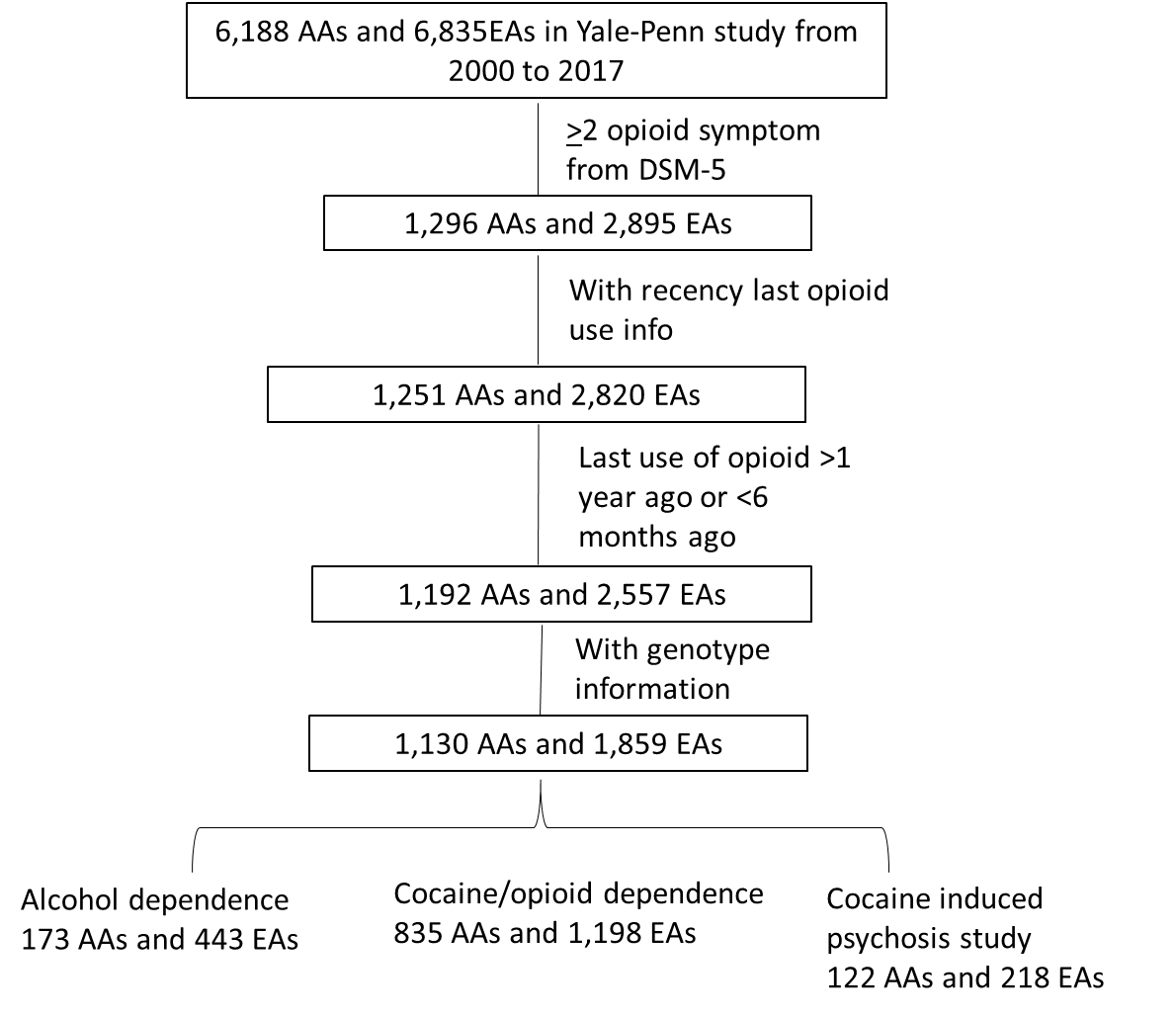


**Supplemental Figure 2**. Predictive accuracy of four machine learning methods by model in African Americans (**A**) and European Americans (**B**). AUC and F1 scores derived from a common set of variables picked by the method (DNN, LASSO, RF, SVM) for each model. m1 = model 1; m2 = model 2; m3 = model 3; DNN = deep neural network; LASSO = least absolute shrinkage and selection operator; RF = random forest; SVM = support vector machine; AUC = area under receiver operating curve; F1 = F1 score.

1. **African Americans** **B. European Americans**


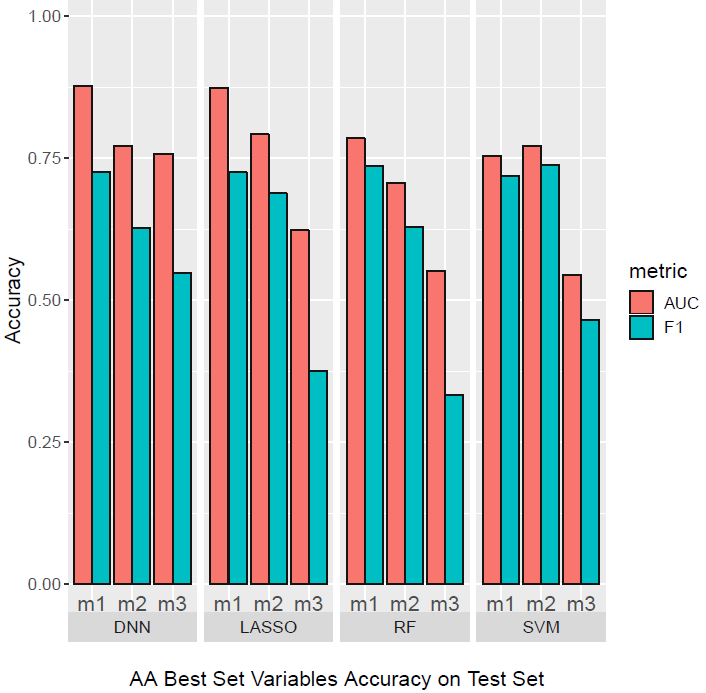

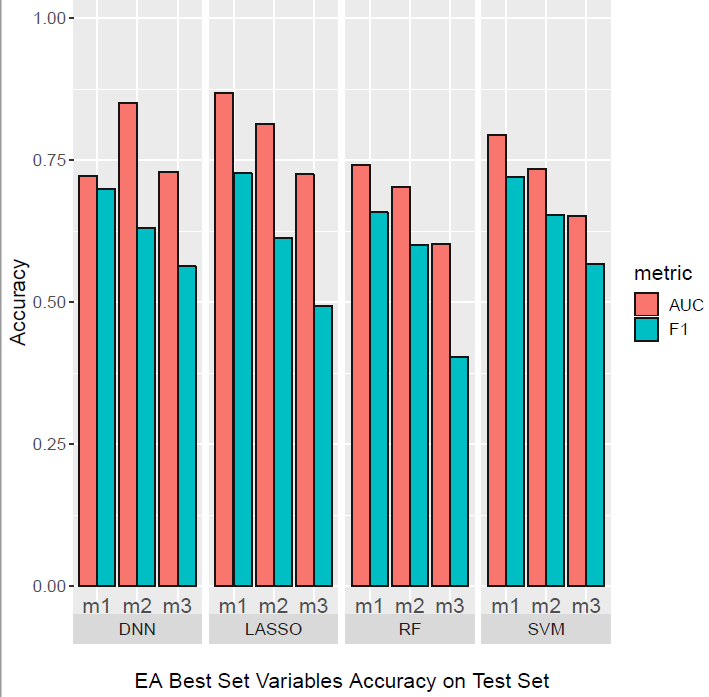


**References**

1. Jerome Friedman TH, Robert Tibshirani. Regularization Paths for Generalized Linear Models via Coordinate Descent. Journal of Statistical Software. 2010;33(1):1-22.

2. Krstajic D, Buturovic LJ, Leahy DE, Thomas S. Cross-validation pitfalls when selecting and assessing regression and classification models. J Cheminform. 2014;6(1):10.

3. Fabian Pedregosa GV, Alexandre Gramfort, Vincent Michel, Bertrand Thirion, Olivier Grisel, Mathieu Blondel, Peter Prettenhofer, Ron Weiss, Vincent Dubourg, Jake Vanderplas, Alexandre Passos, David Cournapeau, Matthieu Brucher, Matthieu Perrot, Édouard Duchesnay. Scikit-learn: Machine Learning in Python. Journal of Machine Learning Research. 2011;12:2825-30.

4. Chih-wei Hsu C-cC, Chih-jen Lin. A practical guide to support vector classification. 2010.

5. Thais Mayumi Oshiro PSP, José Augusto Baranauskas. How Many Trees in a Random Forest? Machine learning and data mining in pattern recognition 8th international conference,. 2012;July.

6. Gilles Louppe LW, Antonio Sutera and Pierre Geurts. Understanding variable importances in forests of randomized trees. NIPS'13 Proceedings of the 26th International Conference on Neural Information Processing Systems. 2013;1:431-9

7. Breiman L. Manual on setting up, using, and understanding random forests v3. 1. Random forests Machine learning, . 2001;45(1):5-32.

8. Chollet F. keras. Github repository. 2015.

9. Diederik P. Kingma JLB. Adam: a method for stochastic optimization. ICLR conference. 2015.

10. Matthieu Courbariaux YB, Jean-Pierre David. BinaryConnect: Training Deep Neural Networks with binary weights during propagations.

11. Adam Paszke AC, Sangpil Kim, Eugenio Culurciello. ENet: A Deep Neural Network Architecture for Real-Time Semantic Segmentation. arXiv. 2016.

12. Chintala ARLMS. Unsupervised representation learning with deep convolutional generative adversarial networks. ICLR conference. 2016.

13. Tieleman TaH, G. Lecture 6.5 - RMSProp, COURSERA: Neural Networks for Machine Learning. Technical report. 2012.

14. Duchi J, Hazan, Elad, and Singer, Yoram. . Adaptive subgradient methods for online learning and stochastic optimization. . The Journal of Machine Learning Research. 2011;12:2121–59.

15. Li Y, Chen CY, Wasserman WW. Deep Feature Selection: Theory and Application to Identify Enhancers and Promoters. J Comput Biol. 2016;23(5):322-36.

16. Chun-Hao Chang LR, Anna Goldenberg. Dropout Feature Ranking for Deep Learning Models. arXiv. 2018;arXiv:1712.08645.

17. Debaditya Roy SRMK, Krishna Mohan Chalavadi. Feature selection using Deep Neural Networks. 2015 International Joint Conference on Neural Networks (IJCNN). 2015.
